# Coronary Computed Tomography Angiography-Derived Modified Duke Index Is Associated with Peri-Coronary Fat Attenuation Index and Predicts Severity of Coronary Inflammation

**DOI:** 10.3390/medicina60050765

**Published:** 2024-05-06

**Authors:** Vasile-Bogdan Halațiu, Imre Benedek, Ioana-Patricia Rodean, Liliana-Oana Cojocariu, Theofana Mihăilă, Emanuel Blîndu, Aurelian Roșca, Botond-Barna Mátyás, Renata Gerculy, Florin Buicu, Theodora Benedek

**Affiliations:** 1 Physiology Department, “George Emil Palade” University of Medicine, Pharmacy, Science and Technology of Târgu Mures, 540139 Târgu Mures, Romania; bogdan.halatiu@umfst.ro; 2 Cardiology Department, Emergency Clinical County Hospital of Târgu Mureș, 540136 Târgu Mureș, Romaniatheofana_m@yahoo.com (T.M.); matyas_botond@yahoo.com (B.-B.M.); gerculy_renata@yahoo.com (R.G.); theodora.benedek@umfst.ro (T.B.); 3 Center of Advanced Research in Multimodality Cardiac Imaging, CardioMed Medical Center, 540124 Târgu Mureș, Romania; 4 Cardiology Department, “George Emil Palade” University of Medicine, Pharmacy, Science and Technology of Târgu Mures, 540139 Târgu Mures, Romania; 5 Doctoral School of Medicine and Pharmacy, “George Emil Palade” University of Medicine, Pharmacy, Science and Technology of Târgu Mures, 540139 Târgu Mures, Romania; 6 Public Health and Health Management Department, “George Emil Palade” University of Medicine, Pharmacy, Science and Technology of Târgu Mures, 540139 Târgu Mures, Romania; florin_buicu@yahoo.com

**Keywords:** CariHeart, cardiac computed tomography, coronary stenosis, fat attenuation index, inflammation

## Abstract

*Background and Objectives*: The modified Duke index derived from coronary computed tomography angiography (CCTA) was designed to predict cardiovascular outcomes based on the severity of coronary stenosis. However, it does not take into consideration the presence or severity of peri-coronary inflammation. The peri-coronary fat attenuation index (FAI) is a novel imaging marker determined by CCTA which reflects the degree of inflammation in the coronary tree in patients with coronary artery disease. To assess the association between the modified Duke index assessed by CCTA, cardiovascular risk factors, and peri-coronary inflammation in the coronary arteries of patients with coronary artery disease. *Materials and Methods*: One hundred seventy-two patients who underwent CCTA for typical angina were assigned into two groups based on the modified Duke index: group 1—patients with low index, ≤3 (n = 107), and group 2—patients with high index, >3 (n = 65). Demographic, clinical, and CCTA data were collected for all patients, and FAI analysis of coronary inflammation was performed. *Results*: Patients with increased values of the modified Duke index were significantly older compared to those with a low index (61.83 ± 9.89 vs. 64.78 ± 8.9; *p* = 0.002). No differences were found between the two groups in terms of gender distribution, hypertension, hypercholesterolemia, or smoking history (all *p* > 0.5). The FAI score was significantly higher in patients from group 2, who presented a significantly higher score of inflammation compared to the patients in group 1, especially at the level of the right coronary artery (FAI score, 20.85 ± 15.80 vs. 14.61 ± 16.66; *p* = 0.01 for the right coronary artery, 13.85 ± 8.04 vs. 10.91 ± 6.5; *p* = 0.01 for the circumflex artery, 13.26 ± 10.18 vs. 11.37 ± 8.84; *p* = 0.2 for the left anterior descending artery). CaRi-Heart^®^ analysis identified a significantly higher risk of future events among patients with a high modified Duke index (34.84% ± 25.86% vs. 16.87% ± 15.80%; *p* < 0.0001). ROC analysis identified a cut-off value of 12.1% of the CaRi-Heart^®^ risk score for predicting a high severity of coronary lesions, with an AUC of 0.69. *Conclusions*: The CT-derived modified Duke index correlates well with local perilesional inflammation as assessed using the FAI score at different levels of the coronary circulation.

## 1. Introduction

Cardiac computed tomography angiography (CCTA) has emerged in the latest years as a first-line option to investigate coronary artery disease (CAD) [1]. Besides providing accurate anatomic information regarding coronary arteries via a non-invasive route, it can reliably identify features of increased vulnerability in the coronary circulation. Various studies investigated the association between these features and different cardiovascular pathologies, such as acute coronary syndromes (ACSs), atrial fibrillation, or heart failure [2,3].

Several CCTA-derived scores have been proposed to characterize the severity of coronary stenoses in an attempt to better stratify the risk of cardiovascular events and restrict invasive coronary imaging to those who really need it. For instance, the modified Duke index derived from CCTA was designed to predict the cardiovascular outcome based on the severity of coronary stenosis. An increased Duke index was proved to be associated with a higher risk of fatal events, validating the role of CCTA as a reliable tool for predicting cardiovascular risk [4].

However, the Duke CAD index does not take into consideration the presence or severity of peri-coronary inflammation. An increased level of inflammation at this level may trigger plaque vulnerabilization, ultimately leading to plaque rupture and acute myocardial infarction [5].

It is well-known that not all unstable plaque progresses to rupture. However, persistently high inflammation at the level of the epicardial fat may lead to plaque progression with high potential for vulnerabilization and rupture in a close future [6,7]. Therefore, assessment of coronary inflammation may have a strong clinical impact, contributing to a superior evaluation of plaque-associated risk. It has been suggested that inflammation may be different at various levels of the coronary circulation, underlining the role of regional flow hemodynamic and shear stress in the complex process of plaque vulnerabilization [8]. In light of this, in addition to the traditional risk factors for CAD, pathologies that cause an increased inflammatory state in the body have recently been identified as risk factors for CAD, emphasizing the major role of inflammation in the progression to ACS. [9,10]. Thus, increased attention should be paid to patients whose associated comorbidities imply an increased inflammatory syndrome.

Nowadays, inflammation may be measured at the level of epicardial fat surrounding coronary arteries using a novel technology patented by Caristo (Oxford, UK) which measures the computed tomography attenuation gradient, which is in direct relationship with the inflammation-mediated change in adipocyte composition and phenotype. This technique measures the peri-coronary fat attenuation index (FAI) validated as a novel imaging marker determined by CCTA which reflects the degree of inflammation in the coronary tree in patients with CAD [11]. A recent analysis published in 2022 indicated that the FAI is associated not only with coronary stenosis, where a stenosis with a diameter ≥50% had significantly higher FAI values compared to those with a diameter <50%, but also with vulnerable plaque features [12].

While the association between the severity of coronary lesions and cardiovascular risk is well-known, there are very few data so far regarding the role of inflammation in the progression of coronary lesions towards a higher severity.

The aim of this study was to investigate the association between two CCTA-derived scores associated with increased cardiovascular risk: peri-coronary inflammation at the level of epicardial fat expressed by CaRi-Heart^®^ analysis and severity of coronary lesions expressed by the Duke CAD index in patients with CAD.

## 2. Materials and Methods

### 2.1. Study Population

This single-center retrospective analysis included 172 adult patients aged over 18 who underwent CCTA at the Center of Advanced Research in Multimodality Cardiac Imaging, Cardiomed Târgu Mureș, Romania, for typical chest pain and a low-to-intermediate likelihood of CAD. Patients with a history of coronary artery disease or non-cardiac chest pain were excluded. Demographic data and risk factors related to coronary artery disease (hypertension, hyperlipidemia, smoking, and diabetes) were obtained prior to the CCTA examination.

Based on the modified Duke index assessed on CCTA, the study population was divided into two groups as follows: group 1—patients with a modified Duke index ≤3 (n = 107), and group 2—patients with a modified Duke index >3 (n = 65).

All the study procedures were carried out in compliance with the Declaration of Helsinki, with the prior approval of the local ethics committee of the Emergency Clinical County Hospital Târgu Mureș (Ad. 26884/10.11.2021) and the George Emil Palade University of Medicine, Pharmacy, Science and Technology Târgu Mureș (1515/09.12.2021).

### 2.2. Coronary Computed Tomography Angiography Acquisition

Image acquisition was performed using a 128-slice high-definition scanner (Siemens Healthcare, Erlangen, Germany) before and after intravenous administration of iodinated contrast according to the patient’s weight (60 to 100 mL). The images were obtained under continuous ECG monitoring in the three bipolar limb leads and after prior determination of blood pressure, heart rate, and O_2_ saturation. Oral metoprolol tartrate was previously administered when needed to achieve a target heart rate below 65 bpm.

### 2.3. Image Analyses

The obtained images were analyzed independently by two randomly selected CCTA interpreters blinded to the clinical data in order to obtain the modified Duke index using a dedicated software (Syngo.via Frontier, Siemens Healthcare, Erlangen, Germany). Based on the atherosclerotic burden, the patients were classified according to the modified Duke index, a coronary plaque severity score for predicting 5-year cardiovascular survival. Calculation of the Duke index has been described previously [4] (Table 1).

All the images were subsequently transferred to the Centre of CARISTO Diagnostics, Oxford, United Kingdom, for assessment of perivascular adipose tissue (PVAT) and related inflammation. Using artificial intelligence algorithms, the PVAT–fat attenuation index (PVAT–FAI) reckons attenuation measurements of perivascular adipose tissue surrounding the coronary arterial wall with high accuracy [13,14]. The FAI HU, FAI score, and FAI score centile were assessed for each of the major coronary arteries. Further, the CaRi-Heart^®^ risk, a score validated to predict a fatal cardiac event within the next 8 years, was assessed for each patient.

### 2.4. Statistical Analysis

The GraphPad InStat 3.10 software (GraphPad Software Inc., San Diego, CA, USA) was used to conduct statistical analysis. Prior to statistical analysis, all the data underwent normality tests. All the data are reported as the absolute values and percentages, or as the medians and standard deviations. For numerical data, unpaired *t*-test or ANOVA test was used for between-group comparisons, and chi-squared test was used for categorical data. Pearson correlation analysis was performed to investigate the association between the modified Duke index and PVAT–FAI. Afterwards, receiver operating characteristic (ROC) analysis was employed to evaluate the ability of the CaRi-Heart^®^ risk to predict a high modified Duke index. The α-value was set at 0.05 for statistical significance.

## 3. Results

### 3.1. Baseline Characteristics of the Study Population

The baseline characteristics of the study population and the differences between the low and high modified Duke index groups are presented in Table 2. The patients with a Duke index > 3 were significantly older (64.78 y.o. vs. 60.03 y.o.; *p* = 0.002) and were more frequently diabetic (36.96% vs. 21.50%; *p* = 0.02). There were no significant differences between the two groups in terms of gender distribution, hypertension, hyperlipidemia, and smoking status (all *p* > 0.05).

### 3.2. Coronary Inflammation and CT-Derived Duke Index

Table 3 summarizes the differences between the two groups in terms of peri-coronary inflammation magnitude and derived risk using standard adipose tissue CT density and percentile curves for the FAI score for each coronary artery analyzed. Based on the standard adipose tissue Hounsfield units (HU), which ranged between −190 to −30 HU, the coronary FAI index values did not differ significantly between the patients with low or high modified Duke index in any of the three coronary arteries analyzed: left anterior descending (LAD), left circumflex (LCX), and right coronary artery (RCA).

**Figure 1:**
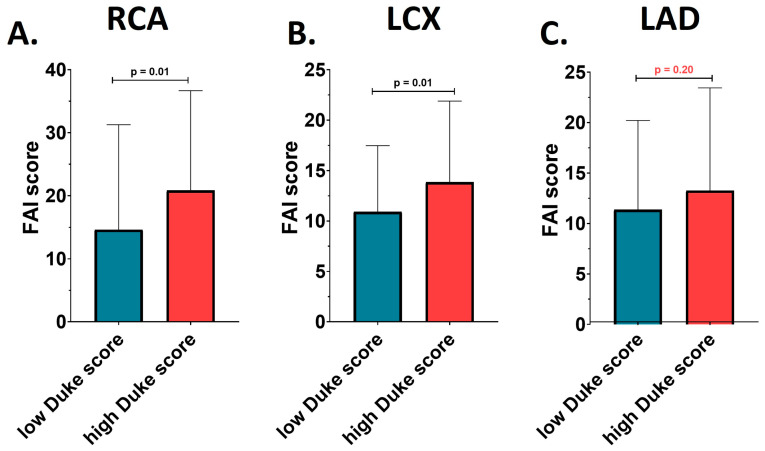
FAI score in the low vs. high modified Duke index patients. (**A**) FAI score at the level of the right coronary artery (RCA). (**B**) FAI score at the level of the left circumflex artery (LCX). (**C**) FAI score at the level of the left anterior descending artery (LAD).

Interestingly, FAI scores of coronary inflammation at the level of the RCA and LCX differed significantly between the two studied groups, while this difference was not observed for the LAD. The patients with a Duke index > 3 presented a significantly higher FAI score at the level of the RCA (20.85 ± 15.83 vs. 14.61 ± 16.66; *p* = 0.02) and LCX (13.85 ± 8.04 vs. 10.91 ± 6.56; *p* = 0.02) compared to the patients with a Duke index ≤ 3, as showed in Figure 1. However, this was not reflected in an increased risk per coronary artery compared with reference standards for the same age group. FAI score percentiles were not significantly different between the two analyzed groups at any of the three coronary arteries analyzed (all *p* > 0.05).

### 3.3. Coronary Inflammation and Risk of Fatal Cardiac Events as Assessed using the CaRi-Heart^®^ Risk Score

**Figure 2:**
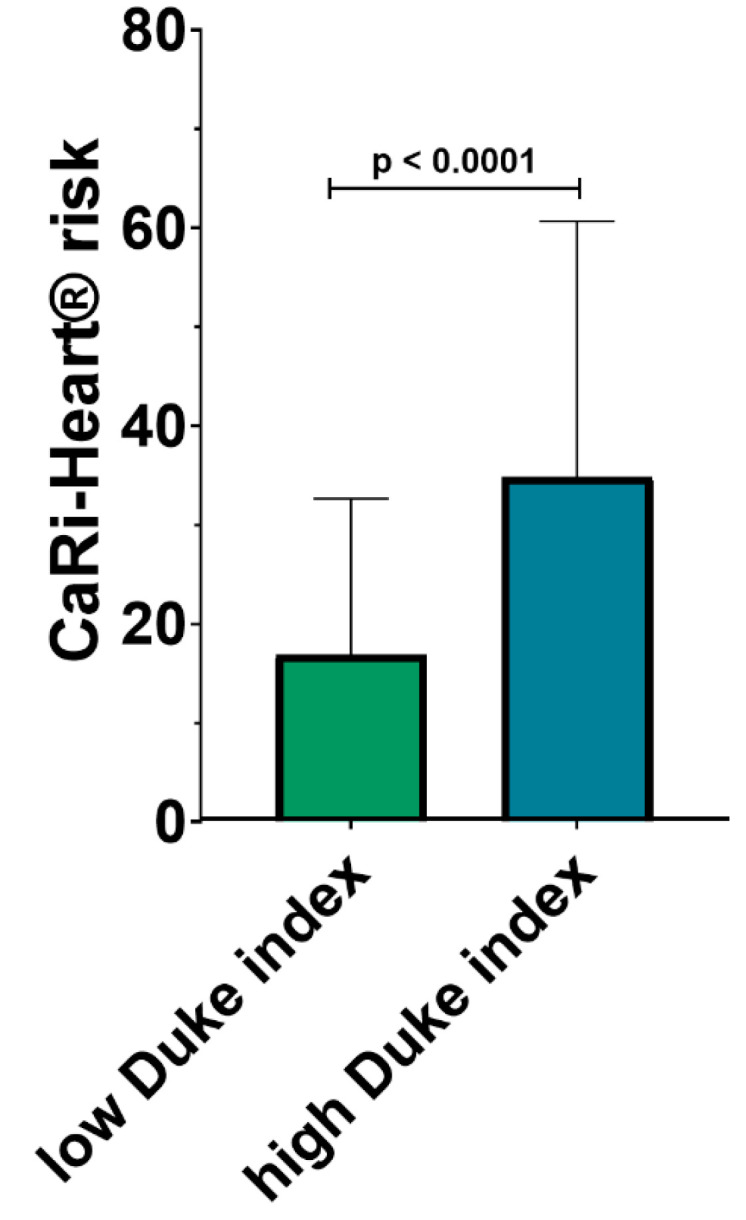
CaRi-Heart^®^ risk in the low vs. high modified Duke index patients.

The risk of future fatal cardiac events was determined using the CaRi-Heart^®^ risk score, integrating the global risk, the individual risks related to inflammation at the level of the three coronary arteries, and the risk factors of the patients (smoker status, hypertension, diabetes, and hypercholesterolemia). CaRi-Heart^®^ analysis identified a significantly higher risk of future events among the patients with a high modified Duke index (34.84% ± 25.86% vs. 16.87% ± 15.80%; *p* < 0.0001) as shown in Figure 2.

**Figure 3:**
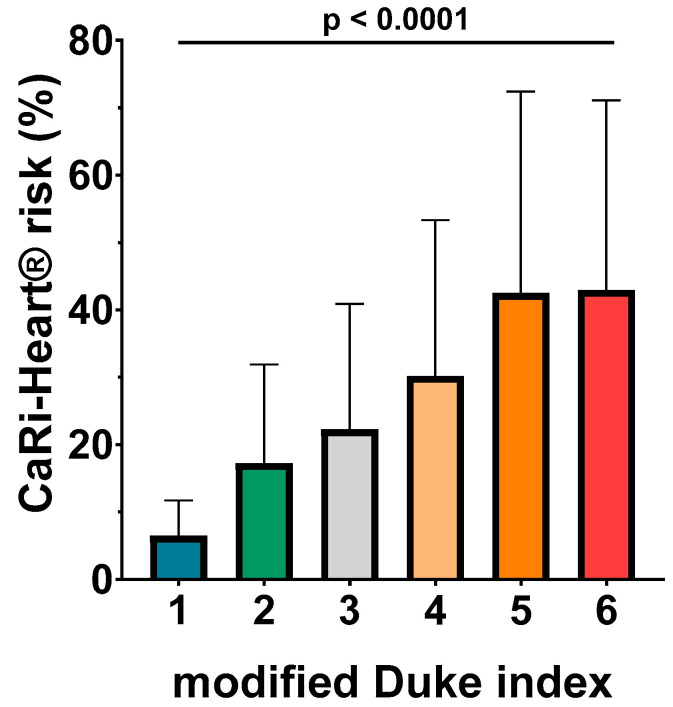
CaRi-Heart^®^ risk in the six classes of the modified Duke index patients.

ANOVA analysis identified a very good association between the CaRi-Heart^®^ risk and the modified Duke index, with higher levels of the CaRi-Heart^®^ score for each superior level of the Duke index (6.46% ± 5.26% for Duke 1; 7.26% ± 14.64% for Duke 2; 22.24% ± 18.65% for Duke 3; 30.15% ± 23.16% for Duke 4; 42.47% ± 29.93% for Duke 5; and 42.95% ± 28.13% for Duke 6; *p* < 0.0001) (Figure 3.).

### 3.4. Correlation between Coronary Inflammation, Cardiac Computed Tomography Lesion Severity, and the CaRi-Heart^®^ Risk

The correlation between the modified Duke index, coronary inflammation, and the CaRi-Heart^®^ risk is presented in Table 4. A high positive correlation was observed between the modified Duke index and the CaRi-Heart^®^ risk as revealed by the Pearson correlation coefficient of 0.75. Weak but significant correlations were also observed between the modified Duke index and the FAI score at the level of all three coronary vessels (all *p* < 0.05).

**Figure 4:**
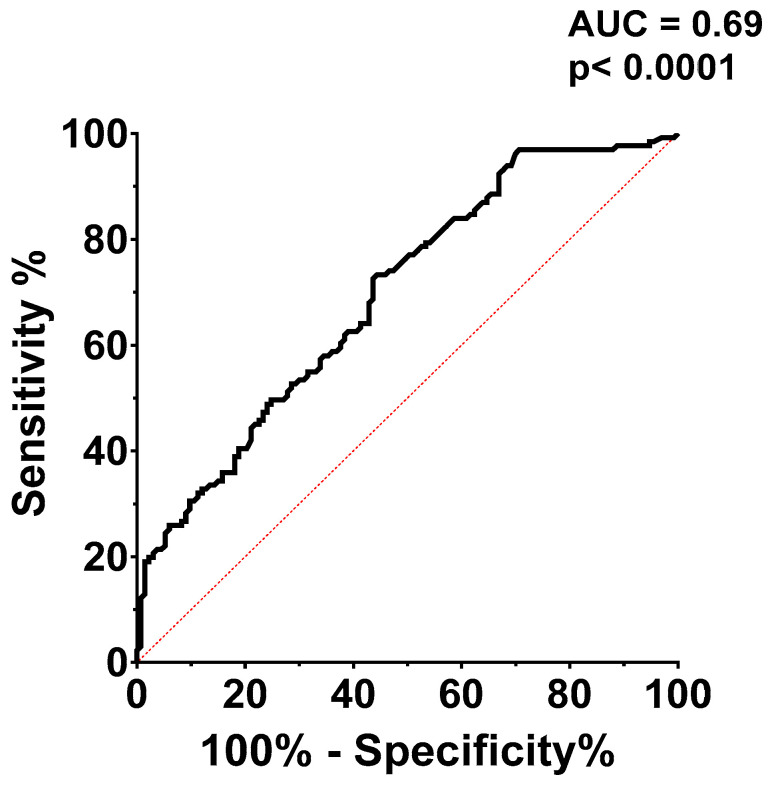
ROC analysis for the accuracy of the CaRi-Heart^®^ risk in predicting a high modified Duke index. AUC = area under the curve.

Receiver operating characteristic (ROC) curve analysis (Figure 4) was used to assess the CaRi-Heart^®^ risk’s ability to predict a modified Duke index above 3. The CaRi-Heart^®^ risk’s optimal cut-off value for predicting a high Duke index was set at 12.1%, with a sensitivity of 61.83% and a specificity of 61.65%, with an AUC of 0.69.

**Figure 5:**
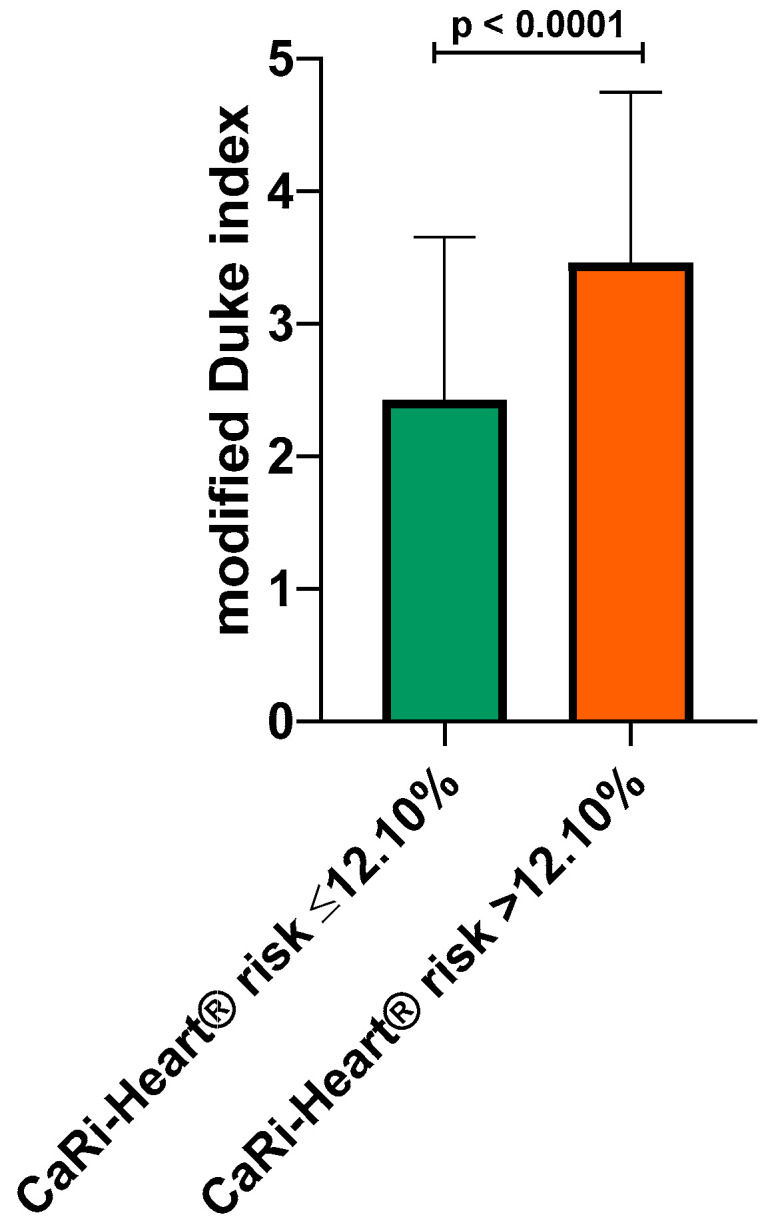
Modified Duke index in the low vs. high CaRi-Heart^®^ risk patients.

Based on this cut-off value, the mean modified Duke index was calculated in the patients with a high CaRi-Heart^®^ risk (below 12.1%) and in the patients with a high CaRi-Heart^®^ risk >12.1%. The mean modified Duke index was significantly higher in the group with CaRi-Heart^®^ risk >12.1% (2.42 ± 1.22 vs. 3.46 ± 1.28; *p* < 0.0001), indicating a very strong association between high inflammation, lesion severity, and future risk of fatal cardiovascular events (Figure 5).

## 4. Discussion

In the present study, we report the results of a CCTA study in patients with a low-to-intermediate likelihood of CAD. Our study is focused on the importance of coronary inflammation in predicting the severity of CAD and indicates that both high inflammatory burden expressed by the FAI index and high CaRi-Heart^®^ risk are associated with coronary lesion severity as revealed by the modified Duke index assessed on the CCTA.

### 4.1. Modified Duke Coronary Artery Disease Index Assessed Using Coronary Computed Tomography Angiography

Over the past ten years, CCTA has become established clinical medicine more than any other non-invasive imaging technique [15]. To identify patients who are at high risk of adverse events, a number of composite risk scores based on CCTA has been proposed lately. The previously reported modified Duke index and prognostic CAD index are used to assess the atherosclerotic burden on CCTA. According to the original Duke index, patients are given a risk score ranging from 0 to 100, which takes into account the severity of stenoses and both the proximal LAD stenosis and the left main stenosis [16]. In a study of 1127 people with a low-to-intermediate risk of CAD, the Duke index was highly correlated with the risk of all-cause mortality [4]. According to data from the ISCHEMIA trial which included over 5000 patients, the severity of ischemia determined by functional tests was not associated with an increased risk of adverse outcomes, while the severity of coronary lesions assessed using the CCTA Duke CAD index was associated with an increased risk of mortality, underlining the significance of this score in the prediction of a poor outcome [17]. A study conducted in China on a population of over 9500 patients with CAD which reported a mortality rate of more than 5% after 4 years found that the risk of death from any cause was proportional to the Duke CAD index [18].

However, the Duke CAD index only includes CCTA-derived information and no information on patients’ risk factors or peri-coronary inflammation. For a better clinical decision and improved outcomes, developing risk scores in combination with the patient’s history and data regarding peri-coronary inflammation are of great importance.

### 4.2. Coronary Inflammation and the Severity of Lesions Expressed by the Duke Index

In the present study, several important findings arise regarding the association between coronary inflammation and the severity of coronary lesions, as indicated by the modified Duke index. Demographic characteristics reveal that individuals with a higher Duke index, indicating more severe coronary lesions, tend to be of older age and have a higher prevalence of diabetes. This aligns with the existing literature, which suggests that both age and diabetes are risk factors of more severe CAD [19,20].

The study’s analysis of peri-coronary inflammation as measured by the FAI using CT imaging reveals nuanced insights. The FAI scores indicative of the degree of inflammation were significantly higher in the RCA and LCX in the patients with a Duke index > 3 compared to those with a lower score. This suggests a correlation between the severity of coronary lesions and the level of inflammation in these specific arteries. However, the LAD did not show this trend, indicating that the relationship between coronary inflammation and lesion severity might be artery-specific, as suggested by previous studies [21,22].

The lack of a significant difference in the FAI score percentiles between the two groups across all three coronary arteries despite the observed differences in the FAI scores suggests that while inflammation is indeed higher in patients with more severe lesions, it may not exceed the expected range for their age group. This could imply that the extent of inflammation in CAD might be more related to individual patient characteristics rather than the severity of the disease itself [23].

The CaRi-Heart^®^ risk score, which integrates the risks associated with coronary arteries and patient-specific risk factors, showed a significantly higher risk of future fatal cardiac events in patients with a high Duke index. This finding underscores the importance of comprehensive risk assessment in patients with CAD considering not just the anatomical severity of the disease, but also the inflammatory milieu and other patient-related factors.

ROC curve analysis for the CaRi-Heart^®^ risk in predicting a high Duke index establishes a specific cut-off value with moderate sensitivity and specificity. This indicates the potential utility of the CaRi-Heart^®^ risk score in clinical settings for predicting lesion severity. Moreover, the significantly higher mean Duke index in the patients with a CaRi-Heart^®^ risk above the cut-off point solidifies the link between higher inflammation, more severe coronary lesions, and an increased risk of fatal cardiac events.

### 4.3. Coronary Inflammation and Cardiovascular Risk

CAD, recognized as the primary contributor to global morbidity and mortality rates [24], is now comprehensively perceived as a process characterized by multiple steps and factors, with chronic inflammation playing a crucial role at each phase [25]. The diverse clinical presentations of CAD, spanning from initial atherosclerotic changes to the advancement of plaques and sudden coronary incidents, highlight the disease’s intricate nature. This underscores the necessity for all-encompassing evaluation methods that prioritize inflammation as the central element, especially in the onset of ACSs via triggering cardioembolic events [26,27].

Recent progress in precision medicine for cardiovascular disease has been marked by significant enhancements in risk assessment methodologies. Contemporary models integrating a range of biomarkers, including those related to inflammation, have improved the accuracy and subtlety of cardiovascular risk predictions. Notably, a link has been established between various inflammatory biomarkers such as fibrinogen, interleukin 6 (IL-6), C-reactive protein (CRP), and galectin-3 and the onset of CVD [28].

A separate meta-analysis investigating the role of vascular inflammation biomarkers in cardiovascular risk prediction further supports this trend. The analysis found that incorporating these biomarkers alongside traditional clinical risk factors significantly improves the prediction of cardiovascular events. This finding accentuates the critical role of inflammation markers in both the evaluation and management of the CVD risk [29]. Additionally, contemporary research is reshaping the understanding of atherosclerosis. New findings challenge the established beliefs and bring to light a range of non-conventional risk factors and intricate biological mechanisms underlying the disease. This evolving perspective emphasizes the complexity of atherosclerosis and the necessity for advanced diagnostic and management strategies in cardiovascular medicine [30].

An emerging focus in cardiovascular research is the measurement of the FAI which carries important clinical implications. Recent investigations have underscored the FAI’s significance as an innovative biomarker for evaluating cardiovascular risk [31,32]. The FAI score, recognized as a proprietary biomarker and a key component of the CaRi-Heart^®^ report, plays a pivotal role in quantifying coronary inflammation. It also serves to gauge an individual’s risk in comparison to a similar demographic group [13].

The development of the peri-coronary FAI as a biomarker for imaging coronary inflammation represents a significant advance in the field. Utilized in conjunction with standard CTA, the FAI plays a crucial role in detecting vulnerable plaques, key indicators for predicting and managing cardiovascular risks [33]. Beyond plaque identification, the FAI’s standardized application in CTA allows for non-invasive monitoring of coronary artery inflammation. This method involves analyzing spatial variations in the composition of perivascular fat, thus offering deeper insights into the inflammatory processes central to CAD [11]. Additionally, this approach enhances prognostic accuracy beyond what traditional risk factors provide, highlighting the critical role of inflammation in comprehensive cardiovascular risk evaluation [34]. Beside inflammation, we should also take into account that CAD displays some differences regarding risk factors and pharmacological treatment, which may influence the outcome [35,36].

The results of the current study must also be seen from the perspective of some limitations. The present study is an observational one; while it can identify associations, establishing a direct cause-and-effect relationship is challenging due to the potential influence of confounding variables. Another limitation of the study is the lack of follow-up data, which would have been of great importance in monitoring the relationship between the imaging markers studied and the cardiovascular outcome.

The present study indicates a correlation between the modified Duke index and perilesional inflammation as assessed by the FAI score. The more pronounced inflammation observed in patients with a modified Duke score >3 should lead to a more aggressive strategy regarding statin treatment as a secondary prevention measure and to reduce mortality in this category of patients. Quantifying both, the modified Duke score and peri-coronary inflammation using the FAI could guide the decision of revascularization strategy for some borderline lesions which present a high degree of inflammation and consequently constitute lesions with an increased vulnerability. The current study emphasizes the additive role of the FAI in the modified Duke score in making an optimal therapeutic decision.

## 5. Conclusions

The CT-derived modified Duke index correlates well with local perilesional inflammation as assessed by the FAI score at different levels of the coronary circulation. The CCTA assessment of coronary inflammation may identify dangerous plaques and reveal increased inflammation in the coronary tree of patients with a low-to-intermediate likelihood of CAD and consequently guide the therapeutic decision.

## Figures and Tables

**Table 1:** Modified Duke prognostic coronary artery disease index based on coronary computed tomography angiography findings [4].

Modified Duke Prognostic CAD Index	CCTA Findings
**1**	Stenosis < 50%
**2**	At least two non-obstructive stenoses (including a coronary artery with proximal stenosis or a stenosis of 50–69%)
**3**	Two coronary arteries with 50–69% stenosis or one coronary artery with 70% stenosis
**4**	Trivascular disease with 50–69% stenoses, or bivascular disease with 70% stenosis, or proximal LAD stenosis of at least 70%
**5**	Trivascular disease with stenoses of at least 70% or bivascular disease with at least 70% stenoses involving proximal LAD
**6**	Left main stenosis of at least 50%

CAD—coronary artery disease; CCTA—coronary computed tomography angiography; LAD—left anterior descending artery.

**Table 2:** Baseline characteristics of the study population.

	All n = 172	Group 1 n = 107	Group 2 n = 65	OR	*p*-Value
**Age (years)**	61.83 ± 9.89	60.03 ± 10.08	64.78 ± 8.90	N/A	0.002 *
**Gender (male)**	119 (69.18%)	70 (58.82%)	49 (41.18%)	0.61	0.17 ^
**Hypertension**	147 (85.46%)	89 (83.18%)	58 (89.23%)	0.59	0.27 ^
**Hyperlipidemia**	90 (52.32%)	54 (50.47%)	37 (56.92%)	0.77	0.43 ^
**Smoker**	31 (18.02%)	17 (15.89%)	14 (21.54%)	0.68	0.34 ^
**Diabetes mellitus**	47 (27.32%)	23 (21.50%)	24 (36.92%)	0.46	**0.02 ^**
**HbA1c (%)**	5.76 ± 0.87	5.70 ± 0.84	5.94 ± 0.82	N/A	0.06 *

The values are expressed as the means ± standard deviation and as absolute values and percentages, respectively; * *p*-values refer to between-group comparisons based on the unpaired *t*-test; ^ *p*-values refer to between-group comparisons based on the chi-squared test. OR—odds ratio.

**Table 3:** PVAT–FAI values in the low and high modified Duke index patients.

		Group 1 n = 107	Group 2 n = 65	*p*-Value
**FAI HU**	**LAD**	−75.31 ± 7.65	−77.23 ± 7.30	0.10
	**LCX**	−70.87 ± 7.28	−71.50 ± 7.88	0.59
	**RCA**	−73.10 ± 9.03	−72.72 ± 9.07	0.79
**FAI score**	**LAD**	11.37 ± 8.84	13.26 ± 10.18	0.20
	**LCX**	10.91 ± 6.56	13.85 ± 8.04	**0.01**
	**RAD**	14.61 ± 16.66	20.85 ± 15.83	**0.01**
**FAI score centile**	**LAD**	0.64 ± 0.27	0.55 ± 0.29	0.06
	**LCX**	0.72 ± 0.24	0.68 ± 0.29	0.29
	**RCA**	0.70 ± 0.29	0.70 ± 0.30	0.97

The values are expressed as the means ± standard deviation; *p*-values refer to between-group comparisons based on the unpaired *t*-test. FAI—fat attenuation index; HU—Hounsfield units; LAD—left anterior descending artery; LCX—left circumflex artery; RCA–right coronary artery.

**Table 4:** Correlations between the modified Duke index and the PVAT–FAI values, score, and the CaRi-Heart^®^ risk.

		*R*	95% Confidence Interval	*p*-Value
**CaRi-Heart^®^ Risk**		0.75	0.70–0.82	**<0.0001**
**FAI HU**	**LAD**	−0.15	−0.29–0.00	**0.04**
	**LCX**	−0.12	−0.26–0.03	0.11
	**RCA**	0.03	−0.11–0.18	0.65
**FAI score**	**LAD**	0.15	0.00–0.29	**0.04**
	**LCX**	0.23	0.08–0.37	**0.002**
	**RAD**	0.19	0.04–0.34	**0.01**
**FAI score centile**	**LAD**	−0.12	−0.27–0.02	0.09
	**LCX**	−0.15	−0.30–0.00	**0.04**
	**RCA**	0.05	−0.09–0.20	0.46

Note: *p*-values refer to Pearson correlation analysis; *R*—Pearson correlation coefficient; FAI—fat attenuation index; HU—Hounsfield units; LAD—left anterior descending artery; LCX—left circumflex artery; RCA—right coronary artery.

## Data Availability

Archived datasets are available upon request by any interested third party.
